# The Effect of Healthcare Worker Density on Maternal Health Service Utilization in Sub-Saharan Africa

**DOI:** 10.4269/ajtmh.21-0727

**Published:** 2022-01-17

**Authors:** Joelle I. Rosser, Kelly Z. Aluri, Arielle Kempinsky, Shannon Richardson, Eran Bendavid

**Affiliations:** Stanford University School of Medicine, Stanford, California

## Abstract

Facility births and antenatal care (ANC) are key to improving maternal health. This study evaluates the relationship between physician and nurse/midwife densities and the use of key maternal health services in sub-Saharan Africa (SSA). We matched individual-level maternal health service indicators from Demographic and Health Surveys between 2008 and 2017, to country-level physician and nurse/midwife per-capita densities, across 35 SSA countries. We performed univariate and multivariate probit regression analyses to evaluate the association between healthcare worker (HCW) densities and facility births as our primary outcome and additional ANC services as secondary outcomes. We controlled for established maternal health predictors, including literacy, child marriage, reported problems accessing healthcare, GDP per capita, political instability, and government effectiveness scores. HCW density across SSA was low at 0.13 physicians and 0.91 nurses/midwives per 1,000 people, compared with 2010 worldwide mean densities of 1.33 and 3.07, respectively. The probability of facility birth increased by 9.8% (95% CI: 2.1–17.5%) for every additional physician per 1,000 people and 8.9% (95% CI: 7.1–9.7%) for every additional nurse/midwife per 1,000 people. HCW densities were also associated with increased likelihood of ANC by the respective provider type, and with antenatal testing for preeclampsia (urine and blood pressure checks). Other ANC services demonstrated variable relationships with HCW densities based on provider type. In 35 SSA countries, HCW density was positively associated with many key measures of maternal health service utilization including facility birth and ANC testing for preeclampsia.

## INTRODUCTION

Sub-Saharan Africa (SSA) has the world’s highest maternal mortality rate, with 546 deaths related to pregnancy or childbirth for every 100,000 live births, compared with only 12 per 100,000 live births in high-income countries.^1^^–^^4^ As of 2017, an estimated 295,000 women die annually because of pregnancy complications, with the majority of those deaths occurring in SSA.^5^ Delivery in a well-equipped healthcare facility and adequate antenatal care (ANC) could contribute to preventing complications and maternal deaths.^1^^,^^6^^,^^7^

The leading causes of maternal mortality in SSA are postpartum hemorrhage and pre-eclampsia/eclampsia.^8^^–^^10^ Multiple studies demonstrate that delivery in a healthcare facility with skilled HCWs who can diagnose and treat these emergencies can significantly lower maternal and neonatal mortality.^9^^–^^11^ However, access to a well-equipped facility is only one determinant of morbidity and mortality and may be insufficient to prevent maternal mortality if the quality of care before, during, and after delivery are inadequate.^12^ High-quality ANC is also key to decreasing maternal mortality. ANC provides an opportunity not only to encourage delivery in a healthcare facility, but also to screen for high-risk conditions such as pre-eclampsia, anemia, and infectious diseases.^13^^,^^14^ Deworming medications, iron and nutritional supplements, and tetanus vaccinations given during ANC visits also decrease the risk of anemia, birth defects, low birth weight, and neonatal tetanus.^13^^,^^15^

Unfortunately, rates of facility births and ANC are both low throughout Africa.^3^ One potential root cause of inadequate maternal health services is the lack of skilled HCWs.^13^ Several studies have shown relationships between HCW density and other health outcome measures including hypertension treatment, overall mortality, life expectancy at birth, infant mortality, under 5 mortality, and maternal mortality.^16^^–^^19^ SSA has the lowest HCW density in the world, which limits the improvements in maternal health that may be possible without expanding the labor supply of skilled providers.^20^ Over the past 15 years, the World Health Report and World Health Organization (WHO) have identified HCW densities that may allow a country to meet a range of healthcare goals.^20^ However, few studies in the past decade have investigated how these densities specifically affect critical maternal health services. Therefore, evaluating the relationship between HCW density and maternal health service utilization is important for plans to augment this workforce, as well as to identify services where HCW density is a critical constraint.

Previous studies on the health implications of HCW density have generally assessed the relationships at an aggregate level and for individual countries.^21^ For example, a study using WHO data estimated HCW density requirements for maternal and child health goals in India.^22^ In addition, although isolated studies examine HCW density in specific countries, no study, to our knowledge, examines the effect of healthcare worker density on maternal health across SSA.^23^

The objective of this study is to evaluate the relationship between HCW density and rates of facility births and ANC visits, diagnostic testing, and treatment across SSA. This is the first study to evaluate the impact of HCW density across SSA using individual-level data on these maternal health outcome measures and controlling for both individual- and country-level covariates.

## METHODS

### Predictor variables: HCW densities.

Data on country-level HCW density was obtained from the World Bank.^24^^,^^25^ The World Bank obtained HCW densities from the WHO’s Global Health Workforce Statistics, Organization for Economic Co-operation and Development (OECD), and supplemental country data. The data from the WHO was compiled from routine administrative information systems (such as public expenditure, staffing, registration, and licensure reports), population censuses, labor force and employment surveys, and health facility assessments.^24^ To allow for comparable health worker data across countries, the WHO used International Labor Organization international standard classification of occupations.^19^

Our primary predictor of interest was HCW density, which included physician density, nurse and midwife density, and a combined HCW density. Physician density per 1,000 included general practitioners and specialized physicians. Nurses and midwives were reported as an aggregate number, because they receive similar training and have overlapping tasks.^19^ A composite measure of provider density, referred to as combined HCWs, was generated that summed physician and nurse/midwife densities.

### Outcome variables: Facility birth and ANC services.

Data on facility birth and ANC services was obtained from the Demographic and Health Survey (DHS) program between 2008 and 2017 for 35 SSA countries. The DHS program collects standardized, nationally representative household and individual survey data across all countries in SSA. We used individual-level data from the standardized DHS “Woman’s Questionnaire,” which includes questions about obstetric care during the most recent pregnancy in women aged 15–49 years.^26^ A complete list of DHS survey questions and how answer choices were categorized in this study is included in Supplemental Table 1.

The primary outcome variable was delivery in a healthcare facility, which we refer to as “facility birth.” This health measure was chosen given its strong negative correlation with maternal mortality.^6^ Additionally, this event was thought to be easiest for mothers to remember and report accurately compared with recall of diagnostic tests performed or antenatal treatments received. Facility birth was defined in the DHS survey as birth of a woman’s most recent child in any of the following: government hospital, provincial/district hospital, health center, health post, other public sector facility, private hospital/clinic, polyclinic, dispensary, or other private sector facility. It does not include women who reported giving birth in their home, another home, on the way to a health facility, or “other.”

Secondary outcome variables included a variety of ANC services including ANC visit with skilled providers; diagnostic tests such as blood pressure and urine checks (testing for pre-eclampsia), blood tests (testing for anemia, HIV, or other infections), and administration of medications such as iron supplements, deworming medications, and tetanus vaccines.

### Covariates.

We controlled for six individual-level covariates included in the DHS survey that are reasonably associated with difficulty accessing skilled maternal healthcare. Four of these factors were reported problems in accessing healthcare during the woman’s most recent pregnancy including could not obtain permission, did not have the monetary funds, distance to facility, and the lack of desire to go alone. The other two factors were literacy (defined as being able to read a complete sentence) and child marriage (defined as first marriage under 18 years old).^27^

We also controlled for three country-level covariates obtained from the World Bank: GDP per capita (reported in thousands USD per capita), political instability, and government effectiveness scores (reported in standard deviation units).^28^^,^^29^ The political instability score is a measure of perceived political instability and potential for politically motivated violence; the government effectiveness score is a measure of the quality of public/civil services and government policies.^29^

### Matching predictor and outcome variables.

Individual observations from the DHS data were matched with the corresponding country-level HCW density and covariates based on country and year. HCW density data were matched to the closest DHS survey year within a 3-year difference.

### Statistical analysis.

We ran univariate and multivariate probit regressions to evaluate the relationship between our predictor variables (i.e., physician density, nurse/midwife density, and composite physician/nurse/midwife density) and outcome variables (i.e., facility birth and ANC services). We did not include a model with both physician and nurse/midwife densities in the same model because there was a strong correlation between nurse/midwife density and physician density (correlation coefficient = 0.815).

We chose to use a probit regression model because it accommodates binary outcome variables and allows for estimation of the average marginal probabilities for each model, providing an easily interpretable result. The marginal probability represents the change in the probability of the outcome in percentage points associated with changes in the main predictors. The mean nurse/midwife density is nearly seven times higher than the mean physician density, so an increase in one provider type is not economically equivalent to the other. However, we present the marginal probabilities for an absolute increase in one physician per 1,000 people or one nurse/midwife per 1,000 people to provide the most intuitive interpretation of change in HCW density. In the supplement, we also present the semi-elasticities of the regression model, which represents the change in outcomes if HCW density were doubled.

In all our analyses, we accounted for sampling stratification and primary sampling units to properly estimate standard errors and confidence intervals. Survey weights were applied using primary sampling unit, strata, and individual weight for each observation, as listed in the DHS survey. Multivariate probit regressions controlled for the individual and country-level covariates listed above. All analyses were performed using Stata IC version 16.1.

## RESULTS

### HCW density.

The mean national nurse/midwife density across all 35 countries in the sample used in this analysis (unweighted) was 0.91 per 1,000 people (range 0.10–5.13), which was nearly seven times higher than physician density, which averaged 0.13 per 1,000 people (range 0.02–0.80). These densities are much lower than the 2010 worldwide nurse/midwife and physician densities of 3.07 and 1.33 per 1,000 people (Table 1).

**Table 1:** Mean HCW densities, DHS survey years, and number of observations by country

Country	Nurse/midwife density*	Physician density*	DHS survey years	Observations
Benin	0.664	0.156	2011, 2012, 2017	13,564
Burkina Faso	0.554	0.046	2010	10,364
Burundi	0.680	0.049	2010, 2011, 2016, 2017	13,576
Cameroon	0.934	0.090	2011	7,628
Chad	0.346	0.046	2014, 2015	11,083
Comoros	0.921	0.170	2012	2,016
Congo, Democratic Republic of	0.470	0.090	2013, 2014	11,279
Congo, Republic of	1.743	0.116	2011, 2012	6,463
Cote d’Ivoire	0.465	0.154	2011, 2012	5,415
Ethiopia	0.202	0.025	2008	7,193
Gambia, The	1.380	0.108	2013	5,375
Ghana	1.387	0.149	2008, 2014	6,440
Kenya	1.292	0.193	2008, 2009, 2014	19,002
Lesotho	0.651	0.068	2009, 2010	3,139
Liberia	0.101	0.037	2013	5,348
Madagascar	0.251	0.182	2008 2009	8,569
Malawi	0.268	0.017	2010, 2015, 2016	27,224
Mali	0.377	0.107	2012, 2013	6,723
Mozambique	0.381	0.051	2011	7,623
Namibia	2.775	0.374	2013	3,968
Niger	0.242	0.051	2012	7,680
Nigeria	1.473	0.380	2008, 2013	38,174
Rwanda	0.736	0.085	2008, 2010, 2011, 2014, 2015	15,548
Senegal	0.676	0.147	2010–2017	34,843
Sierra Leone	0.730	0.022	2008, 2013	12,504
South Africa	5.132	0.802	2016	3,036
Tanzania	0.339	0.026	2009, 2010, 2015, 2016	12,408
Togo	0.298	0.049	2013, 2014	5,012
Uganda	0.849	0.099	2011, 2016	15,172
Zambia	0.786	0.163	2013, 2014	9,351
Zimbabwe	1.175	0.073	2010, 2011, 2015	9,230

DHS = Demographic Health Survey. Mean physician density and nurse/midwife densities for each country during that period are given in columns 2 and 3. The years for which DHS survey data were available between 2008 and 2017 and the total number of individual observations included in the final analysis for each country are given in columns 4 and 5.

* Mean provider density as a ratio per 1,000 people

### Maternal health outcomes.

The final analysis included 367,184 women from the included countries, surveyed between 2007 and 2018. About 59.7% of the women reported a facility birth for their most recent pregnancy. Additionally, 62.4% reported ANC from a nurse or midwife, 12.6% reported ANC from a physician, and overall 67.5% of women reported receiving ANC from any skilled provider. Of the ANC services reported blood pressure check had the highest rate at 86.0% and antiparasitic treatment had the lowest rate at 38.9% (Table 2).

**Table 2:** Rates of maternal health service uptake in the study population

Maternal health service	Percentage of women	Observations in univariate analysis	Observations in multivariate analysis
Delivery in a healthcare facility	59.7	344,950	293,968
ANC from any skilled provider	67.5	344,950	293,968
ANC from a doctor	12.4	343,479	293,376
ANC from a nurse or midwife	62.0	343,479	293,376
Antenatal blood pressure check	86.0	299,996	262,175
Antenatal urine sample	63.2	299,835	262,058
Antenatal blood sample	80.1	299,869	262,068
Antenatal iron supplements	76.6	335,239	292,268
Antenatal anti-parasitic treatment	38.9	318,851	278,418
Any tetanus vaccine during pregnancy	77.6	344,950	293,968
Two tetanus vaccines during pregnancy	54.1	344,950	293,968
Antenatal HIV test	66.2	206,636	191,863

ANC = antenatal care. The percentage of women across all countries and DHS surveys included in this study who reported receiving each maternal health service during their most recent pregnancy are described above. Additionally, the number of individual observations included in univariate and multivariate analysis is provided for each outcome measure. All multivariable analyses included at least 85% of the observations.

### Association between HCW density and facility birth.

In our multivariable regression models, physician density, nurse/midwife density, and combined HCW density were all positively associated with facility birth. The likelihood of a facility birth increased by 9.8% (95% CI: 2.1–17.5%, *P* = 0.015) for every additional physician per 1,000 people, 8.9% (95% CI: 7.1–9.7%, *P* < 0.001) for every additional nurse/midwife per 1,000 people, and 9.0% (95% CI: 7.6–10.4%, *P* < 0.001) for every additional HCW in either profession (Table 3). Using elasticities, the likelihood of facility birth increased by 1.2% and 6.6% for a doubling of physician and nurse/midwife densities, respectively (Supplemental Table 2). Doubling the combined number of HCWs was associated with a 7.7% increase in facility birth.

**Table 3:** Marginal probabilities of maternal health service by medical provider density (per 1,000 people)

	Nurse/Midwife				Physician				Combined healthcare workers (physicians + nurses/midwives)			
	Unadjusted		Adjusted		Unadjusted		Adjusted		Unadjusted		Adjusted	
	MP (%)	95% CI	MP (%)	95% CI	MP (%)	95% CI	MP (%)	95% CI	MP (%)	95% CI	MP (%)	95% CI
Facility birth	5.45**	(4.6, 6.3)	8.89**	(7.1, 9.7)	−11.67**	(−16.5, −6.8)	9.81*	(2.1, 17.5)	3.41**	(2.7, 4.1)	9.02**	(7.6, 10.4)
ANC from skilled provider	4.53**	(3.7, 5.3)	7.54**	(5.7, 9.3)	0.54	(−3.9, 5)	−19.49**	(−28.4, −10.6)	3.05**	(2.4, 3.7)	2.14**	(0.6, 3.7)
ANC from a doctor	4.48**	(4.1, 4.8)	0.63	(−0.1, 1.3)	2.9**	(27.2, 31.1)	34.76**	(31.1, 38.4)	4.25**	(3.9, 4.6)	1.51**	(0.9, 2.1)
ANC from a nurse or midwife	2.10**	(1.4, 2.8)	3.92**	(2.2, 5.6)	−12.09**	(−16.2, −8)	−45.64**	(−54.3, −37)	0.73*	(0.1, 1.3)	−1.53*	(−3, 0)
Antenatal blood pressure check	9.63**	(9, 10.2)	1.14*	(0.1, 2.1)	39.2**	(36.1, 42.4)	4.22	(−1.7, 10.2)	7.50**	(7.0, 8.0)	−0.17	(−1.1, 0.8)
Antenatal urine sample	26.20**	(25.3, 27.1)	10.53**	(8.8, 12.3)	103.97**	(99.5, 108.4)	19.83**	(10.9, 28.8)	21.51**	(20.7, 22.3)	8.55**	(6.9, 10.2)
Antenatal blood sample	13.79**	(12.9, 14.7)	13.9**	(12.7, 15.1)	16.11**	(13.1, 19.1)	−31.96**	(−38.7, −25.2)	9.84**	(9.1, 10.5)	9.69**	(8.6, 10.7)
Antenatal iron supplements	0.18	(−0.4, 0.8)	−4.44**	(−5.4, −3.5)	−17.74**	(−21.4, −14.1)	−12.87**	(−19, −6.7)	−1.07**	(−1.6, −0.5)	−5.38**	(−6.3, −4.4)
Antenatal anti-parasitic treatment	−10.45**	(−11.5, −9.4)	−0.44	(−2.1, 1.2)	−77.85	(−81.3, −74.4)	−16.43**	(−25.2, −7.7)	−11.82**	(−12.7, −11)	−4.51**	(−6.1, −3)
Any tetanus vaccine during pregnancy	−2.15**	(−2.7, −1.6)	1.35*	(0.5, 2.2)	−33.13	(−36.5, −29.8)	−22.41**	(−27.7, −17.1)	−3.14**	(−3.6, −2.7)	−.51**	(−2.3, −0.7)
Two tetanus vaccines during pregnancy	−2.22**	(−2.8, −1.7)	−4.10**	(−5.3, −2.9)	−23.70**	(−27.1, −20.3)	4.57	(−1.6, 10.9)	−3.47**	(−4, −3)	−8.37**	(−9.4, −7.3)
Antenatal HIV test	5.87**	(5, 6.7)	15.39**	(13.7, 7.1)	−23.09**	(−27.4, −18.7)	−50.39**	(−58.8, −42)	3.17**	(2.5, 3.9)	8.10**	(6.5, 9.7)

ANC = antenatal care; CI = confidence interval; MP = marginal probability. The marginal probabilities for outcomes measures are provided above for increasing nurse/midwife density or physician density by one individual per 1,000 people in the population for univariate and multivariate analyses.

* *P* < 0.05; ** *P* < 0.01.

### Association between HCW density and ANC.

The likelihood of receiving ANC by a physician increased by 34.8% (95% CI: 31.1–38.4%) with every additional one physician per 1,000 people in multivariable regression. The likelihood of receiving ANC by a nurse/midwife increased by 3.9% (95% CI: 2.2–5.6%) for every additional nurse/midwife per 1,000 people. Increased physician density was negatively associated with receiving ANC from a nurse/midwife, whereas increased nurse/midwife density was marginally positively associated with receiving ANC from a physician (Table 3).

There was a positive association between HCW densities and diagnostic testing for preeclampsia. There was a strong and consistent association between provider densities and urine checks. In multivariate regression, the likelihood of a urine check during ANC increased by 10.5% (95% CI: 8.8–12.3%) for every additional nurse/midwife and increased by 19.8% (95% CI: 10.9–28.8%) for every additional physician per 1,000 people. The increased likelihood of a blood pressure check was more modest at 1.1% (95% CI: 0.1–2.1%) for nurse/midwife density.

The relationship between other ANC services and provider density was more mixed. The marginal probabilities of having a blood sample collected, an HIV test, and at least one tetanus vaccine were positively associated with higher nurse/midwife density but negatively associated with physician density. Both physician density and nurse/midwife density were either not significantly associated or were negatively associated with iron supplementation and anti-parasitic drugs in both univariate and multivariate analyses. The relationships between outcome measures and the composite HCW density generally mirrored that of nurse/midwife density.

## DISCUSSION

In this investigation of the relationship between HCW densities and utilization of maternal health services in 35 SSA countries, we found that overall HCW density is extremely low in SSA. The WHO’s Ending Preventable Maternal Health initiative identified a target minimum HCW density of 5.9 per 1,000 people to decrease maternal deaths to 50 per 100,000 lives births.^20^ However, our study shows an average of only 0.91 nurses/midwives and only 0.13 physicians per 1,000 people, numbers far below the WHO recommendation.

Our study provides a unique contribution because it is the first study to our knowledge that evaluates HCW density and maternal health utilization across SSA using individual-level data on maternal health outcome measures and covariates. Our analysis demonstrates that increased HCW density is associated with an increased likelihood of facility birth. Every additional nurse/midwife per 1,000 was associated with an 8.9% increased chance of facility birth; whereas every additional physician per 1,000 was associated with a 9.8% increased chance of facility birth. Every additional HCW in either profession was associated with a 9.0% increased probability of facility birth, although this combined HCW variable was heavily weighted by nurses and midwives that are more abundant in all countries. Increased physician and nurse/midwife densities were also associated with an increased likelihood of receiving ANC by a physician or nurse/midwife, respectively. The relationship between HCW density and specific ANC services however was more variable.

The variable association between HCW density and specific ANC services raises interesting questions about the role of physicians and nurses in distributing healthcare resources. Recall about specific ANC services may be poor compared with remembering whether delivery occurred in a health facility or whether any ANC care was received; therefore, these specific ANC variables may be less accurate.^30^^–^^32^ However, it is also possible that different types of healthcare providers tend to provide different ANC services. For instance, screening for preeclampsia may be more likely to be administered by a skilled provider; this hypothesis is consistent with our data showing a strong association between HCW density and urine tests. In contrast, other ANC services such as HIV testing, deworming, and iron and vaccine distribution may be more likely to be administered in mass health campaigns or by other types of HCWs not included in this analysis. It is also plausible that places with fewer physicians and nurse/midwives have more robust community health worker (CHW) programs or programs to conduct mass campaigns that reach a larger number of women than individual-level care. Given the specific ANC services that are associated with HCW density, it would be interesting to further investigate whether these differences have an impact on clinical outcomes, such as maternal mortality and rates of complications such as hemorrhage and eclampsia.

Our study has limitations that should be acknowledged. First, we used DHS survey data that is susceptible to recall bias. More detailed questions, such as the skill level of the ANC provider or the specific services received, are more likely to be subject to this bias. However, even several years later, a woman would likely be able to accurately recall our primary outcome, whether they delivered in a health facility. It seems unlikely that the rate of inaccurate reporting would differ significantly based on HCW density and so we assume any bias would be nondifferential.

Second, our study does not take into account ANC received by other types of HCWs such as community health workers (CHWs). CHWs are frequently an important part of the healthcare system in low-income countries and may play a larger role in ANC provision in places where physician or nurse/midwife density is lower. CHWs may also play a key role in large community health campaigns, such as for HIV testing or antiparasitic drugs administration. Unfortunately, CHW training rigor can be highly variable and high-quality, publicly available data on CHW density is sparse. This makes it difficult to evaluate the contribution of these other HCWs to maternal healthcare.

In addition to density of CHW’s, our model is unable to adjust for other possible unobserved confounders such as health insurance systems or nongovernmental organization involvement in the maternal health service provision. We do adjust for significant confounders identified in previous studies, including illiteracy, child marriage, and distance to a healthcare facility.^1^^,^^7^^,^^33^^–^^35^ We also adjust for GDP, political instability, and government effectiveness scores, which we assume serve as reasonable proxies for some unmeasured confounders including national healthcare expenditure and quality of health insurance systems. Additionally, we adjust at the individual level for questions from the DHS survey about problems accessing medical care.

Finally, there are limitations in the HCW density measure. HCW density is reported at the country level and does not account for differential healthcare access within a country. HCW densities are only measured once every several years for individual countries and do not necessarily align exactly with the year of maternal health service delivery. However, the process for collecting the HCW densities is through labor accounting methods and is unlikely to be related to the outcomes of interest. Any error in the measurement of HCW density, therefore, could lead to attenuation bias in our estimate. Additionally, within country effects could not be evaluated because many countries did not have repeated measures of HCW density within our time frame. Despite these limitations, measurement errors should be nondifferential and should not bias the results. Additionally, the countries with repeat measures demonstrated relatively stable HCW densities over time.

Our primary outcome of interest—facilities births—should be relatively robust to these potential problems and is consistent with country-level studies showing a relationship between HCW density and facility birth.^7^ Our study provides supportive evidence that increasing the healthcare workforce in SSA may be important for improving maternal child health. However, the magnitude of the increase needed to make these gains and the relatively importance of provider type much be carefully considered. The likelihood of a facility birth increased by 8.8% and 9.6% for every additional nurse/midwife and physician per 1,000 people, respectively. Such an increase would mean more than doubling of nurse/midwife density and increasing physician density by more than 8-fold, on average. Further research is needed to characterize the relative importance of and optimal ratios of different types of providers, including physicians, nurse/midwives, and CHWs.

Furthermore, although facility birth generally improves maternal outcomes, that relationship depends heavily on the environment, quality of care, and resources available at the facility.^36^^–^^38^ Thus, increased HCW numbers is only one of many critical components to improving maternal health care in SSA. Further investigation is required to better understand how to balance adequate investment in the HCWs with other determinants of maternal health.

## Supplemental Material


Supplemental materials
