# Supplementary material for: The Effect of Healthcare Worker Density on Maternal Health Service Utilization in Sub-Saharan Africa
Source: Am J Trop Med Hyg. 2022 Jan 17;106(3):939–44. doi: 10.4269/ajtmh.21-0727 (PMC8922518; doi:10.4269/ajtmh.21-0727)
Supplement: Supplementary file 1 [file tpmd210727.SD1.pdf]

Supplemental Table 1. DHS questions used for maternal health indictors and individual covariates

Variable	DHS Survey Question	Answers Categorized as Yes	Answers Categorized as No	Answers Considered Missing*
**Primary Outcome**				
Facility birth: delivered in a skilled facility the last time they gave birth	Where did you give birth to (name of youngest child under the age of 5 years)?	Government hospital<br>Government health center<br>Government health post<br>Other public sector<br>Private hospital/clinic<br>Other private sector	Her home<br>Other home	Missing
**Antenatal Care Provider**				
Received ANC** from a physician	(1) Did you see anyone for antenatal care for this pregnancy?<br>(2) Whom did you see?	(1) Yes<br>**AND**<br>(2) Doctor	(1) No<br>**OR**<br>(2) Nurse/Midwife<br>Auxiliary midwife<br>Traditional birth attendant<br>Village health worker	Missing either (1) or (2)
Received ANC from a nurse or midwife	(1) Did you see anyone for antenatal care for this pregnancy?<br>(2) Whom did you see?	(1) Yes<br>**AND**<br>(2) Nurse/Midwife	(1) No<br>**OR**<br>(2) Doctor<br>Auxiliary midwife<br>Traditional birth attendant<br>Village health worker	Missing either (1) or (2)
**Services and Treatment during ANC**				
Blood pressure measured during ANC	As part of your antenatal care during this pregnancy, was your blood pressure measured at least once?	Yes	No	Missing
Urine sample taken during ANC	As part of your antenatal care during this pregnancy, did you give a urine sample at least once?	Yes	No	Missing
Blood sample taken during ANC	As part of your antenatal care during this pregnancy, did you give a blood sample at least once?	Yes	No	Missing
Received HIV test during ANC	I don't want to know the results, but were you tested for HIV as part of your antenatal care?	Yes	No	Missing
At least one tetanus shot during ANC	(1) During this pregnancy, were you given an injection in the arm to prevent the baby from getting tetanus, that is, convulsions after birth?<br>(2) During this pregnancy, how many times did you get a tetanus injection?	(1) Yes<br>**AND**<br>(2) 1, 2, or Don't Know	(1) No	(1) Missing<br>Don't know
Two tetanus shots during ANC	(1) During this pregnancy, were you given an injection in the arm to prevent the baby from getting tetanus, that is, convulsions after birth?<br>(2) During this pregnancy, how many times did	(1) Yes<br>**AND**<br>(2) 2	(1) No<br>**OR**<br>(2) 1, Don't Know	(1) Missing<br>Don't know

	you get a tetanus injection?			
Took iron during pregnancy	During this pregnancy, were you given or did you buy any iron tablets or iron syrup?<br>(Sample tablets and syrup were shown to the participant.)	Yes	No	Missing<br>Don't know
Took anti-parasitic drugs during pregnancy	During this pregnancy, did you take any drug for intestinal worms?	Yes	No	Missing<br>Don't know
**Individual covariates**				
Difficulty accessing medical advice or treatment because… (following 4 rows)	Many different factors can prevent women from getting medical advice or treatment for themselves. When you are sick and want to get medical advice or treatment, is each of the following a big problem or not a big problem?			
… Getting permission to go to the doctor	Getting permission to go to the doctor?	Big problem	Not a big problem	Missing
… Getting money needed for advice or treatment	Getting money needed for advice or treatment?	Big problem	Not a big problem	Missing
… The distance to the health facility	The distance to the health facility?	Big problem	Not a big problem	Missing
… Not wanting to go alone?	Not wanting to go alone?	Big problem	Not a big problem	Missing
Literate	Now I would like you to read this sentence to me.<br>*Participant is provided with a card with one sentence*	Able to read whole sentence	Cannot read at all<br>Able to read only part of the sentence	No card with required language<br>Blind<br>Visually impaired<br>Missing
Child marriage	(1) Are you currently married or living together with a man as if married?<br>(2) Have you ever been married or lived together with a man as if married?<br>(3) How old were you when you first started living with him?	[(1) Yes **OR** (2) Yes]<br>**AND**<br>(3) Younger than 18	(1) No **AND** (2) No<br>**OR**<br>(3) 18 or older	(1) **AND** (2) Missing **OR** (3) Missing

*Not all questions had "Don't know" as an option. For those that did, "Don't know" answers were considered missing.
**ANC = antenatal care
Questions obtained from the DHS Program Model Questionnaires, Phase 7 (1)

Supplemental Table 2. Change in maternal health service uptake when HCW density (per 1,000 people) doubles

	Nurse/Midwife				Physician			
	Unadjusted		Adjusted		Unadjusted		Adjusted	
	Elasticities (%)	95% Confidence Interval	Elasticities (%)	95% Confidence Interval	Elasticities (%)	95% Confidence Interval	Elasticities (%)	95% Confidence Interval
Facility birth	4.26**	(3.6, 4.9)	6.64**	(5.4, 7.4)	-1.57**	(-2.2, -0.9)	1.20*	(0.3, 2.2)
ANC from skilled provider	3.51**	(3.3, 4.4)	5.65**	(6, 8.5)	0.07	(-0.5, 0.6)	-2.45**	(-3.3, -1)
ANC from a doctor	4.10**	(3.8, 4.5)	0.57	(0.1, 1.3)	4.73**	(4.3, 5.1)	5.45**	(4.8, 6)
ANC from a nurse or midwife	1.67**	(1.5, 2.6)	3.00**	(3.6, 6.1)	-1.62**	(-2.2, -1.1)	-5.75**	(-6.6, -4.4)
Antenatal blood pressure check	5.96**	(5, 5.6)	0.71*	(-1.6, -0.3)	3.80**	(3.6, 4.1)	0.39	(-0.6, 0.6)
Antenatal urine sample	16.89**	(15.2, 16.3)	6.76**	(2.8, 4.9)	10.52**	(10.2, 11)	1.88**	(0.6, 2.3)
Antenatal blood sample	8.64**	(8.1, 9)	8.71**	(7.6, 8.9)	1.86**	(1.5, 2.2)	-3.40**	(-4, -2.6)
Antenatal iron supplements	0.15	(-0.3, 0.6)	-3.53**	(-3.8, -2.3)	-2.45**	(-2.9, -1.9)	-1.74**	(-2.5, -0.9)
Antenatal anti-parasitic treatment	-7.64**	(-8, -6.6)	-0.32	(0.4, 2.6)	-9.02**	(-9.4, -8.7)	-1.82**	(-2.7, -0.7)
Any tetanus vaccine during pregnancy	-1.78**	(-2.2, -1.3)	1.10**	(0.9, 2.2)	-4.84**	(-5.3, -4.3)	-3.17**	(-3.8, -2.3)
2 tetanus vaccines during pregnancy	-1.80**	(-2.2, -1.3)	-3.19**	(-3.3, -1.5)	-3.16**	(-3.6, -2.7)	0.58	(-0.1, 1.4)
Antenatal HIV test	4.47**	(3.8, 5.1)	11.37**	(10.2, 12.6)	-2.94**	(-3.5, -2.3)	-6.07**	(-7.1, -5.1)

	Combined healthcare workers (physicians + nurses/midwives)			
	Unadjusted		Adjusted	
	Elasticities (%)	95% Confidence Interval	Elasticities (%)	95% Confidence Interval
Facility birth	3.1**	(2.4, 3.7)	7.7**	(6.5, 8.8)
ANC from skilled provider	2.9**	(2.3, 3.5)	3.3**	(2, 4.6)
ANC from a doctor	4.5**	(4.2, 4.9)	1.7**	(1.0, 2.3)
ANC from a nurse or midwife	0.9**	(0.3, 1.4)	0.3	(-1.0, 1.6)
Antenatal blood pressure check	5.0**	(4.6, 5.3)	-1.4**	(-2.1, -0.7)
Antenatal urine sample	15.1**	(14.5, 15.6)	4.1**	(2.9, 5.3)
Antenatal blood sample	7.1**	(6.7, 7.5)	7.0**	(6.3, 7.7)
Antenatal iron supplements	-1**	(-1.5, -0.5)	-4.6**	(-5.5, -3.8)
Antenatal anti-parasitic treatment	-9.5**	(-10.1, -8.9)	-2.2**	(-3.4, -0.9)
Any tetanus vaccine during pregnancy	-3.0**	(-3.4, -2.5)	-1.0**	(-1.7, -0.3)
2 tetanus vaccines during pregnancy	-3.2**	(-3.6, -2.5)	-6.7**	(-7.6, -5.8)
Antenatal HIV test	2.9**	(2.4, 3.7)	7.5**	(7.0, 7.9)

Abbreviations: ANC = Antenatal care. *p<0.05; **p<0.01. Combined healthcare workers includes both physician and nurse/midwifes.
The elasticities from univariate and multivariable probit regressions are provided above. These represent the percent increased likelihood a woman would receive maternal health services given a doubling of nurse/midwife and physician densities.
